# Effect of Fibre-Enriched Orange Juice on Postprandial Glycaemic Response and Satiety in Healthy Individuals: An Acute, Randomised, Placebo-Controlled, Double-Blind, Crossover Study

**DOI:** 10.3390/nu11123014

**Published:** 2019-12-10

**Authors:** Neus Bosch-Sierra, Roger Marqués-Cardete, Aránzazu Gurrea-Martínez, Carmen Grau-Del Valle, Carlos Morillas, Antonio Hernández-Mijares, Celia Bañuls

**Affiliations:** 1 Service of Endocrinology, University Hospital Doctor Peset, Foundation for the Promotion of Health and Biomedical Research in the Valencian Region (FISABIO), Avda Gaspar Aguilar 90, 46017 Valencia, Spain; neusboschsi@gmail.com (N.B.-S.); carmendosaguas@hotmail.com (C.G.-D.V.); carlos.morillas@uv.es (C.M.); hernandez_antmij@gva.es (A.H.-M.); 2 Zumos Valencianos del Mediterraneo S.A., Calle del Pollancar (Pol Industrial Parc Sagunt I) S/N, 46520 Puerto de Sagunto, Valencia, Spain; rmarques@zuvamesa.com (R.M.-C.); agurrea@zuvamesa.com (A.G.-M.); 3 Department of Medicine, University of Valencia, Avda de Blasco Ibañez 15, 46010 Valencia, Spain

**Keywords:** citrus fibre, orange juice, glycaemia, insulin, satiety, gut hormones, healthy subjects

## Abstract

*Background*: Consumption of fibre-enriched orange juice may be an appropriate way to supplement daily fibre intake and achieve beneficial effects on metabolic health. The present study aimed to assess the short-term effects of fibre-enriched orange juice on postprandial metabolism and satiety in a healthy adult population. *Methods*: In this double-blind, randomised, placebo-controlled, crossover study 10 healthy subjects underwent two one-day trials in which they consumed an orange juice beverage containing 1.4 g/100 mL of citrus fibre (29.3% soluble and 41.9% insoluble) or a placebo (regular orange juice without added fibre). Postprandial glucose, insulin, gut hormones (GLP1, GIP and ghrelin), leptin and qualitative appetite/satiety assessment were measured every 15 or 30 min over the 120 min test period. *Results*: The fibre-enriched orange juice decreased postprandial serum glucose and circulating insulin levels at 15 min compared with the placebo. In addition, after intake of the fibre-enriched juice, a significant effect on qualitative feelings of satiety and fullness was observed at 15 and 120 min, and was accompanied by a significant decrease in GLP1 response at 15 min. No significant changes were observed in leptin, GIP and ghrelin after juice intake. *Conclusions*: In healthy individuals, a single acute consumption of fibre-enriched orange juice has short-term beneficial effects on postprandial glycaemia, circulating insulin levels and satiety through GLP1 secretion.

## 1. Introduction

Dietary fibre is the edible portion of plant-derived foods that are resistant to enzymatic digestion and absorption in the gut [1]. The health benefits associated with dietary fibre are multiple; for example, on gastrointestinal (GI) health and a reduction of the risk of various diseases including cardiovascular diseases, diabetes type 2, colorectal cancer and pathologies derived from obesity, such as metabolic syndrome [2].

According to major food and health organisations, the recommended total dietary fibre intake in adults is at least 25 g per day, and should include soluble and insoluble fibre [3]. However, the average intake of dietary fibre is lower than recommended, at around 20 g/day in the European population [3], due to a low consumption of fruits, vegetables, legumes and cereals. In this context, fibre supplements added to the regular diet could be the key to achieving the recommended intake.

Fibre consumption is associated with the feeling of satiety, and thus with prospective food intake [4]. Consequently, lack of satiety is a key conditioner of the caloric intake of individuals and is crucial to the high prevalence of obesity worldwide. Therefore, it is important to understand how the intake of different nutrients or foods can influence it [5]. The effect on the feeling of satiety can vary depending on the physicochemical properties (hydration, viscosity, particle size, fermentability) and type of fibre, and this can have an impact on the caloric intake of individuals. In this way, fibre contributes substantially to energy balance [4].

Among the different food matrices, drinks and beverages are a suitable method by which to supplement fibre intake, soluble fibre being the most employed [1]. Several studies have demonstrated an association between the consumption of viscous fibre-enriched beverages and increased satiety [6], with a dose-dependent effect being reported [7,8]. The mechanism of action underlying these effects could be a slowing of gastric emptying and intestinal transit, which would reduce intestinal glucose absorption and modulate the secretion of GI hormones involved in the regulation of appetite [9]. The consumption of soluble dietary fibre has been shown by different studies to increase the production of plasma glucagon-like peptide 1 (GLP1) and peptide YY (PYY), depending on the dose, usually when greater than 5 g [10,11,12,13,14]. In addition, the intake of soluble fibre can have an effect on ghrelin levels, decreasing their secretion in a healthy adult population [12,14,15]. 

On the other hand, the beneficial effect of dietary fibre consumption has received substantial attention due to its well-known potential to form viscous solutions that reduce the glycaemic response [16]. It has been proposed that high viscous fibre can lead to a decrease in postprandial glycaemia and insulin response [17], while the effect of soluble fibre on fasting glucose and insulin levels has been demonstrated in overweight adults [18]. Several acute and chronic studies have used fruit juice as a matrix for fibre enrichment, obtaining remarkable effects on the postprandial glycaemic response of both healthy subjects and men with metabolic risk when doses of fibre were moderately high [19,20,21,22]. However, there is little evidence of the effect of dietary fibre intake on glycaemic profile, satiety and the secretion of GI hormones on a healthy adult population in the case of non-sweetened fruit juice enriched with an extract of the fruit’s own fibre (albedo and flavedo obtained from the orange peel), a novel by-product of orange juice production composed of different types of soluble and insoluble fibre. 

Therefore, in the present study, we evaluate the effectiveness of dietary fibre intake both subjectively, by means of behavioural markers and a visual analogue scale (VAS), and objectively, using physiological markers such as serum gut hormone levels. In this way, we set out to investigate the acute effects of fibre-enriched orange juice, on the postprandial glycaemic response, feeling of satiety and the secretion of hormones that regulate appetite in a healthy adult population.

## 2. Material and methods

### 2.1. Subjects

A total of 10 healthy adult volunteers were recruited among university students, laboratory and clinical staff and relatives at the outpatient endocrinology service of the University Hospital Dr Peset between January and April 2019. 

The inclusion criteria were age 18–45 years, a moderately active lifestyle, and body mass index (BMI) of 20–25 kg/m^2^. Exclusion criteria were pregnancy or lactation, fasting glycaemia > 100 mg/dL on at least two previous occasions, diabetes, or medication known to interfere with glucose metabolism.

The study was carried out according to the guidelines of the Declaration of Helsinki, and all procedures involving human subjects were approved by the Ethics Committee of the University Hospital Doctor Peset (CEIm:89/18). All participants signed a written informed consent before participating in the trial. None of the ten originally enrolled volunteers withdrew from the study due to personal reasons, because of an inability or unwillingness to comply with the protocol.

### 2.2. Study design

A randomised, double-blind, placebo-controlled, cross-over study was performed. 

The participants were randomly assigned to placebo or fibre-enriched orange juice groups at baseline using a standardized computer programme and cross-over; in this way, each subject completed two 1-day trials separated by an interval of 1–2 weeks between the two trials. 

Prior to initiation of the study, fasting blood glucose was measured in order to exclude subjects with fasting glycaemia > 100 mg/dL. In addition, participants received dietary guidelines from an experienced dietitian in order to homogenise food consumption and, thus, reduce within-subject-variability and interference with the glycaemic response. The subjects were recommended to choose a standard meal (1500–2500 Kcal) that complied and to consume it 24 h before each intervention. Alcohol and beverages containing caffeine were ruled out and subjects were encouraged to continue their usual physical activities in the days prior to the interventions, but to avoid intense activity. 

A 24 h diet recall (compiled on the day before a trial) was performed by a dietitian, and food intake was converted into energy and nutrient values using the web tool Odimet_®_, in order to assess the calorie and macronutrient intakes of our sample. The diet recalls revealed no deviations from the guidelines during the course of the study.

### 2.3. Orange Juice Production

The fibre-enriched product and the placebo, suitably prepared in their intervention food matrix (250 mL PET containers), were ingested (drunk in this case) within a time interval of approximately 5 min. The enriched product contained a minimum of 3.5 g of total fibre and was served in a volume of 250 mL, with a total of 25.5 g of carbohydrates. The placebo was a commercially available juice with a similar composition to the functional juice, but without added fibre. The nutritional composition of the commercial orange juice used as a matrix (placebo) and the juice enriched in fibre is detailed in Table 1. Both the functional juice and placebo were prepared by Zumos Valencianos del Mediterráneo S.A. in their selected dietary matrix, and were sent appropriately coded and packaged in opaque labelled bottles to the hospital to enable blind masking. Participants and the investigators who performed sample and data analyses were completely blind. The samples were refrigerated until administration. Before ingestion, the beverages were shaken vigorously for 1 min and the subjects were advised to consume them in less than five minutes. 

In the case of orange juice with fibre, 100% orange juice (not from concentrate) was analysed based on sensory and technological nutritional aspects to establish the percentage of citrus fibre. We developed a fibre-enriched orange juice with organoleptic properties as close as possible to those of a commercial orange juice, including taste (absence of bitter notes and off-flavours), aroma (absence of strange notes/off-flavours) and mouthfeel (viscosity). Maintaining these sensorial aspects was one of the main reasons for adding 1.4 g/100 mL of fibre to the juice; larger amounts significantly changed the sensorial profile. When designing the drink, other aspects were taken into account to guarantee a low impact on viscosity, such as the selection of fibre particles and the homogenization process. In addition, to prevent differences at a visual level, the container used for both products had a label that obscured the content inside. Organoleptic evaluation was performed by experienced testers following the internal methodology of the International Federation of Fruit Juice Producers (IFU Analysis No. 25, 2005). The content of 1.4 g fibre/100 mL (3.5 per 250 mL) allowed the guarantee of a sensorial profile similar to commercial orange juices on the market. The origin of the fibre was orange albedo and flavedo (orange peel). The process consisted of crushing the orange peel, followed by scalding and centrifugation, plus a drying step to finally proceed to its micronization. Dietary fibre was determined by the enzymatic–gravimetric method AOAC 985.29. Firstly, we determined the insoluble and total fibre, and then we calculated the soluble by difference. In brief, enzymatic attack with alpha-amylase, protease and amyloglucosidase was performed, followed by soluble fibre precipitation (only for total fibre content) and determination of fibre by gravimetry (after deducting proteins and ashes from the residue). The nutritional composition of the fibre used to make the fibre-enriched juice is described in Table 2.

The juice with fibre and the placebo were subjected to ultra-high pressure homogenization (UHPH) (Micro DeBee, BeeInternational). Subsequently, the juice was pasteurized in order to extend the shelf-life of the product for the duration of the study. Juices were stored at 4 °C in a dry and well-ventilated environment until consumption.

The UHPH treatment allowed the homogenization of the fibre in the juice. The sensory tests determined that treatment with 100 MPa was the most adequate for this application since treatment with higher pressure provided a sensorially acceptable but excessive viscosity.

### 2.4. Blood Sampling, Anthropometrical and Biochemical Measurements

After 12-h overnight fasting, a catheter was inserted into the participant’s antecubital vein, and a blood sample was collected at baseline (minute 0; while still fasting), and subsequently at 15, 30, 45, 60, 90 and 120 min after consumption of the fibre-enriched juice or placebo. Blood samples were collected in Vacutainer serum separator tubes (BD, Franklin Lakes, NJ) for glucose, insulin and GI hormone analysis. Blood samples were allowed to clot (30 min) and were then centrifuged at 2000× *g* for 15 min at 4 °C, and glucose was measured in the freshly separated serum. The remaining aliquots of serum were stored at –80 °C until they were used to analyse insulin and GI hormones.

Regarding anthropometrical parameters, weight was determined using electronic scales, height was measured with a stadiometer, waist and hip circumference were measured using a metric tape and BMI was calculated by dividing weight in kilograms by the square of height in metres. Blood pressure was measured twice consecutively using a mercury sphygmomanometer, with approximations of 0.5 cm.

Glucose concentrations were determined by the enzymatic method of hexokinase in a Beckman LX-20 autoanalyser (Beckman Coulter, La Brea, CA), with an intra-assay coefficient of variation < 3.5%. Insulin concentrations were determined by an enzyme-linked immunosorbent assay (human insulin ELISA, Millipore Corporation, Billerica, MA; intra-assay coefficient of variation < 5%). The calibration curve was linear within the concentration range of 2–200 μU/mL. The integrated area under the curve (AUC) for glucose and insulin (at 120 min) was calculated using the trapezoidal method.

The effect on appetite/satiety was studied according to different serum hormonal markers (GLP1, ghrelin, leptin, gastric inhibitory polypeptide (GIP)) using Luminex X-MAP technology at 0, 15, 30 and 45 min. In addition, an assessment of satiety and hunger was carried out using a test of 5 questions about appetite and a VAS score, consisting of lines of 100 mm anchored at each end with opposite statements (minimum “0” and maximum “100”). The subjects placed an “X” on the line to indicate their sensation of satiety, hunger, desire to eat, fullness and prospective consumption (“0” for no sensation and “100” for maximum sensation). The score was calculated by measuring the distance in millimetres from the beginning of the line to the position of the “X” (from left to right) for five questions: (1) Satiety: how great is your feeling of fullness?, (2) Hunger: how hungry do you feel?, (3) Desire to eat: how intense is your desire to eat?, (4) Fullness: how full do you feel?, and (5) Prospective consumption: how much food do you think you are capable of eating or would you like to eat now? This assessment was carried out concomitantly to the blood extractions performed at baseline (immediately before intake), and 15 (immediately after intake), 60 and 120 min after ingestion.

### 2.5. Statistical Analyses

Based on our preliminary data, sample size was 10 subjects per group, in order to provide 80% statistical power and, thus, detect differences between the two paired groups in the values of the primary efficacy criterion (postprandial glycaemia variation) equal to or greater than 5 mg/dL, assuming a common standard deviation of 5 mg/dL.

Statistical analysis was carried out with the statistics program SPSS version 17.0 (SPSS Inc., Chicago, IL). Data are expressed as mean standard error of the mean (SEM). Shapiro–Wilk tests were used to assess normality for continuous variables, and data conformed to normal distribution patterns. Differences within groups were analysed using two-factor repeated-measures analysis of variance (ANOVA). A Bonferroni correction was used for the post hoc multiple comparisons. Therefore, differences in AUC between placebo and fibre-enriched juice groups were analysed using a paired Student’s *t*-test. All tests employed a confidence interval of 95%, and differences were considered significant when *p* < 0.05.

## 3. Results

A total of 10 healthy individuals were recruited, of whom 50% were men. The mean age of the participants was 29.6 ± 5.8 years. The anthropometric characteristics of the subjects are shown in Table 3.

**Figure 1:**
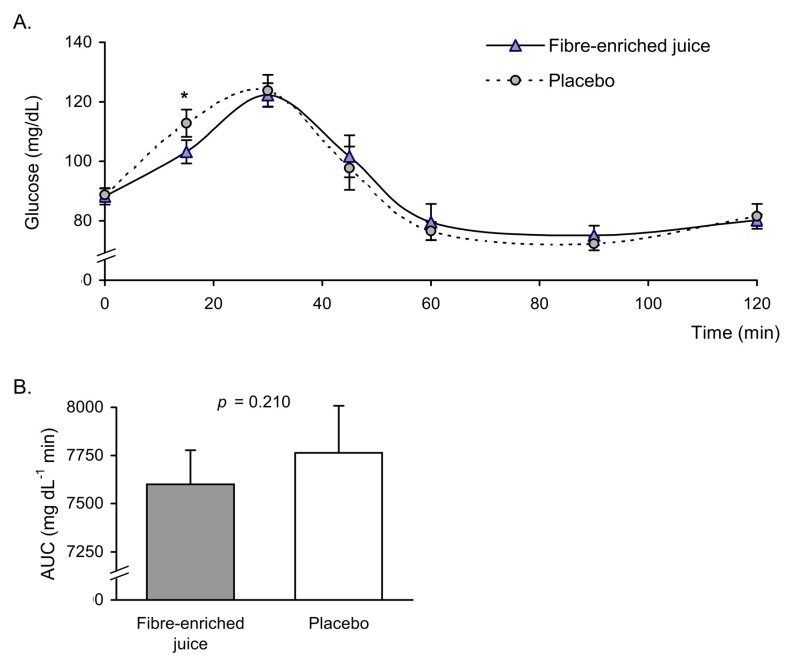
(**A**) Glucose levels (mg/dL) at baseline and after oral ingestion of orange juice enriched in fibre and its respective placebo. (**B**) Area under the curve (AUC) of glucose (mg dL^−1^min) in fibre-enriched orange juice and placebo groups. Data are represented as mean ± standard error (*n* = 10 in each group). Glucose: time by group interaction *p* = 0.297; * *p* < 0.05 when compared by two-factor repeated-measures analysis of variance (ANOVA) followed by post hoc multiple comparisons. Differences in AUC between placebo and fibre-enriched juice groups were analysed using a paired Student’s *t*-test.

**Figure 2:**
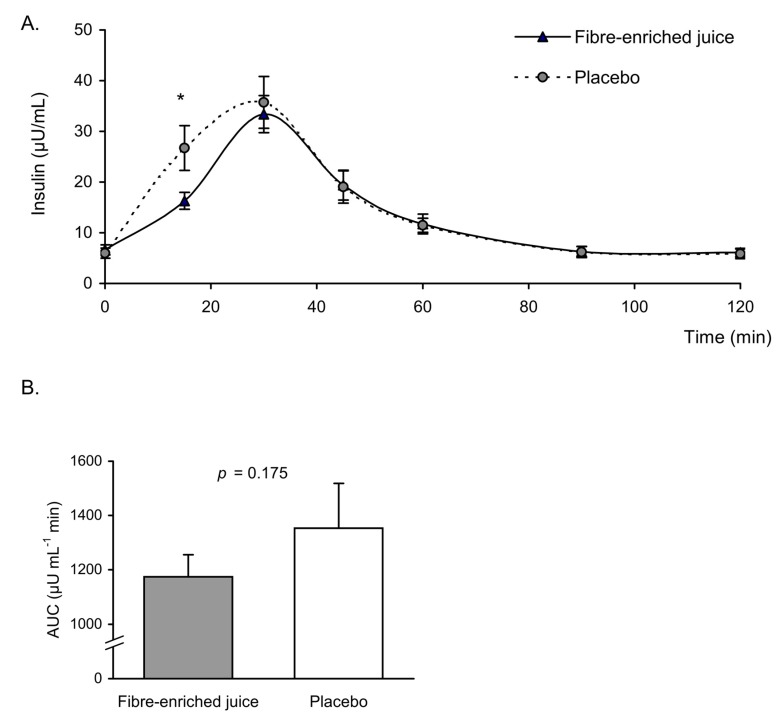
(**A**) Insulin levels (μU/mL) at baseline and after oral ingestion of orange juice enriched in fibre and its respective placebo. (**B**) Area under the curve (AUC) of insulin (μU mL^−1^ min) in fibre-enriched orange juice and placebo groups. Data are represented as mean ± standard error (*n* = 10 in each group). Insulin: time by group interaction *p* = 0.149; * *p* < 0.05 when compared by two-factor repeated-measures analysis of variance (ANOVA) followed by post hoc multiple comparisons. Differences in AUC between placebo and fibre-enriched juice groups were analysed using a paired Student’s *t*-test.

Regarding the glycaemic curves, we observed significant differences 15 min after ingestion of the product, at which point blood glucose levels were lower compared to those in the placebo group (Figure 1A). The area under the glucose curve did not show significant differences in the group that ingested the enriched juice (*p* = 0.210) (Figure 1B). A simultaneous increase in insulin was observed in the placebo group (Figure 2A). The area under the insulin curve showed a downward trend in the group that ingested the enriched juice, but differences between the two beverages were not significant (*p* = 0.175) (Figure 2B).

**Figure 3:**
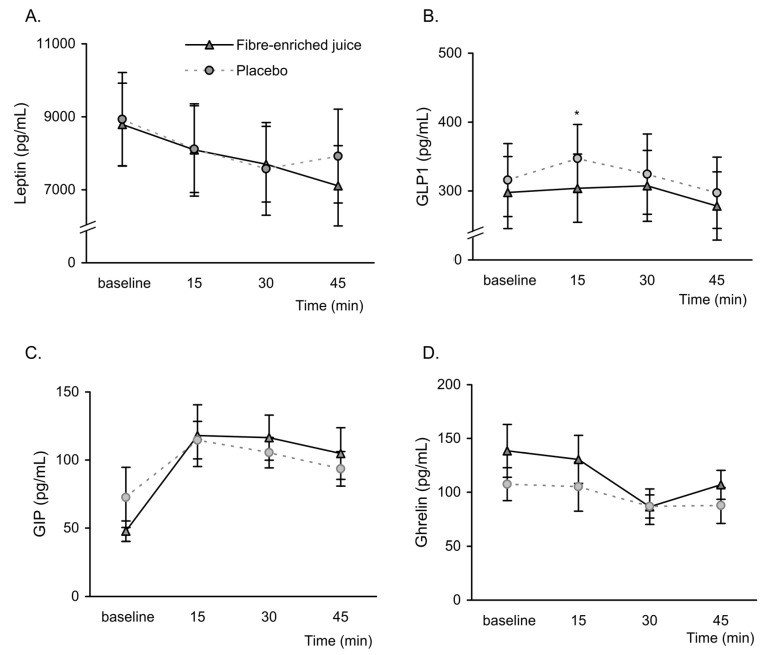
Concentration of gastrointestinal peptides (pg/mL) at baseline and after oral ingestion of orange juice enriched in fibre and its respective placebo. (**A**) Leptin, (**B**) GLP1, (**C**) GIP, (**D**) ghrelin. Data are represented as mean ± standard error (*n* =10 in each group). Leptin: time by group interaction *p* = 0.368; GLP1: time by group interaction *p* = 0.200; GIP: time by group interaction *p* = 0.308; Ghrelin: time by group interaction *p* = 0.607; * *p*.< 0.05 when compared by two-factor repeated-measures analysis of variance (ANOVA) followed by post hoc multiple comparisons.

With respect to gastrointestinal peptides, leptin showed a gradual decrease over time after the ingestion of both the enriched product and the placebo, with the former showing a downward trend with respect to the latter, which was trending towards significance (*p* = 0.08) at 45 min (Figure 3). In addition, GLP1 showed a slightly significant increase after the ingestion of the placebo at 15 min, whereas it remained fairly stable after that of the fibre-enriched juice. Ghrelin, although manifesting a pronounced drop 30 min after ingestion of the enriched product, showed no significant differences with respect to the placebo. Similarly, GIP did not show changes between the products under study.

**Figure 4:**
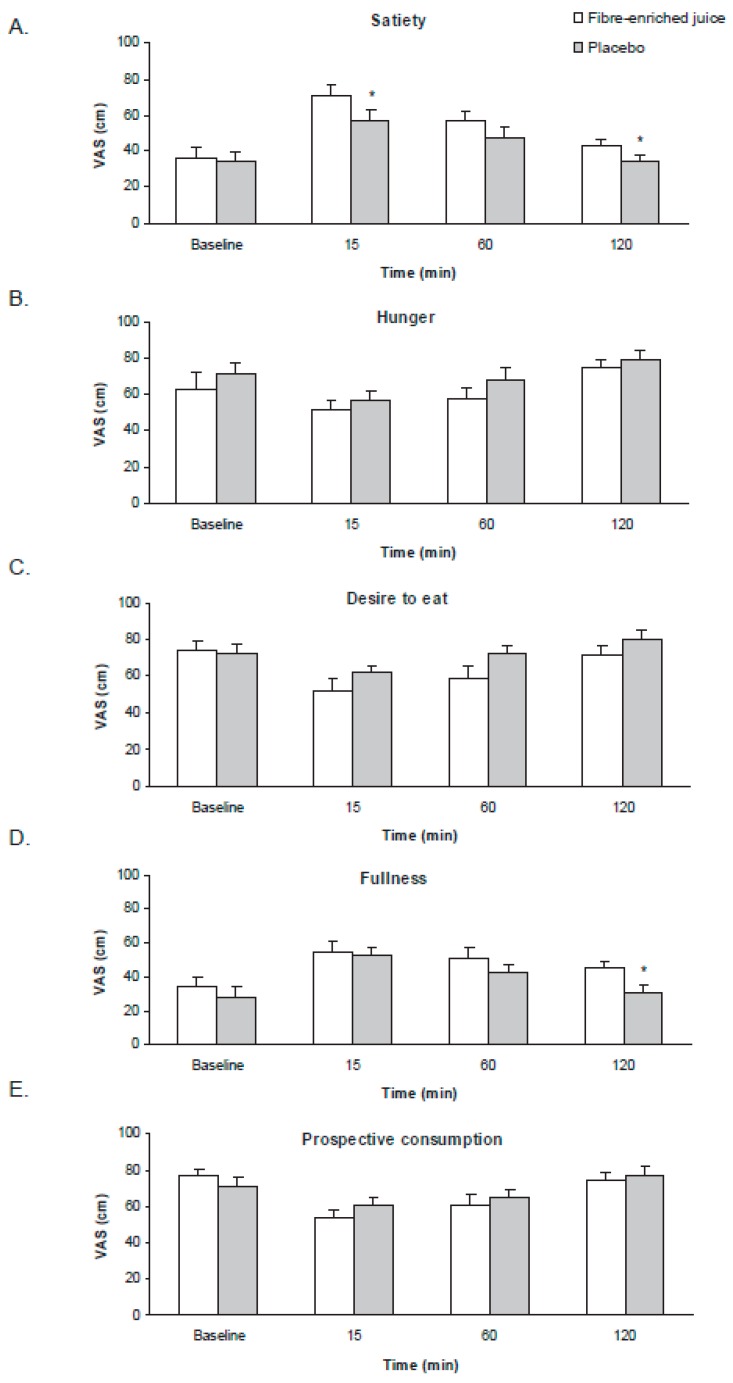
Satiety assessment before and after oral ingestion of fibre-enriched orange juice and its respective placebo using a visual analogue scale. Data corresponding to the visual analogue scale used to evaluate (**A**) satiety, (**B**) hunger, (**C**) desire to eat, (**D**) fullness, and (**E**) prospective consumption. Data are represented as mean ± standard error (*n* = 10 in each group). Satiety: time by group interaction *p* = 0.398; hunger: time by group interaction *p* = 0.833; desire to eat: time by group interaction *p* = 0.332; fullness: time by group interaction *p* = 0.196; prospective consumption: time by group interaction *p* = 0.232; * *p* < 0.05 when compared by two-factor repeated-measures analysis of variance (ANOVA) followed by post hoc multiple comparisons

The questionnaires referring to subjective appetite ratings-measured by VAS scores revealed significant differences between the beverages. It is noteworthy that the question relating to satiety showed significant changes at 15 and 120 min, with higher values in the product versus placebo group (Figure 4). The fullness score was also significantly different at 120 min in the enriched product group compared to the placebo group. The feeling of hunger, desire to eat, and prospective consumption did not show significant differences.

## 4. Discussion 

This study shows that the consumption of fibre-enriched orange juice (3.5 g per beverage) modifies the time course of glucose and insulin responses. There is an attenuation of the postprandial glycaemic response of both glucose and insulin levels early on after juice intake with respect to a placebo, though the impact on overall glucose and insulin response curves is negligible. In addition, we have seen that consumption of a fibre-enriched beverage enhances feelings of satiety and fullness (at the beginning and the end of the experimental period) and that this is accompanied by an alteration of GLP1 secretion.

The effect of dietary fibre on satiety regulation, due to the capacity of certain types of fibre to produce viscous solutions, is well-demonstrated [23]. Viscous fibres form gels in the stomach during food digestion, increasing stomach distension, which in turn triggers signals of fullness through the afferent vagus nerve system [6,23]. Studies comparing the effect of high vs. low viscous fibre preloads show that high viscosity increases satiety and modifies prospective intake in healthy subjects [24,25,26,27]. The effect of viscous fibre enrichment and satiety has been studied mainly in solid food matrices such as cereals, with a positive association in different types of populations (adults at cardiometabolic risk and healthy groups) usually been reported [28,29,30], though other studies have failed to find an effect [31,32,33]. In contrast, fibre-enriched liquid matrices such as fruit juices have been found to produce a significantly increased sensation of satiety in healthy and slightly overweight adults [8,13,14]. 

In the present study, we have observed significant differences in postprandial VAS after fibre-enriched orange juice intake, including a significant increase in satiety and fullness sensation. This result is consistent with those of other studies using fibre-enriched fruit juices at a greater dose than 3 g [7]. Nonetheless, we observed significant differences between fibre-enriched orange juice and placebo groups in satiety at 15 min, and both satiety and fullness sensation at 120 min. This could be due to an early effect of the fibre and its viscosity in the stomach during the first 15 min after its ingestion, with no effect present after 45 min, in accordance with the results of the gut hormone response. We assessed only short-term hormone secretion, while other groups have detected significant postprandial differences in gut hormone secretion > 90 min after consuming the fibre-enriched product [13,14]. Thus, we cannot be sure if the significant difference at 120 min was due to hormone secretion or other factors. We did not find an association between orange pomace enrichment and hunger or prospective eating in a short-term intervention, as reported by other groups [14], even when a higher dose of total fibre was administered. This indicates there are other factors that may modulate satiety regulation, including the type of fibre, its viscosity, the baseline characteristics of subjects, and the matrix itself, and would explain the different results observed between studies. Furthermore, we did not evaluate prospective consumption with an objective measure (such as a meal) after the intervention, so our results may not be comparable to those of other studies. 

Intestinal satiety differs from gastric satiety in that it is nutrient-dependent, not mechanical. Viscous fibres prolong gut transit time and the absorption rate of nutrients like monosaccharides, affecting the release of satiety-regulating hormones (CCK, GLP1, PYY) and, in turn, satiety and hunger nucleus signalling by the central nervous system [6,34]. In addition, GLP1 stimulates insulin secretion after a meal in order to ensure glucose homeostasis [35]. The effect of fibre supplements and their viscosity on postprandial glucose response is widely known [16] and is attributed to two mechanisms: the hindering of glucose absorption in the intestinal mucosa and the regulation of glucose homeostasis through GLP1 postprandial releasing. Several interventional studies have shown that different types of fibre enrichment attenuate glucose and insulin postprandial responses, increase secretion of anorexigenic gut hormones such as PYY, and reduce the secretion of orexigenic hormones like ghrelin in healthy and overweight adults [13,14,22,31,32,36]. Contradictory effects on GLP1 postprandial levels have been reported [28], and little is known about the effect of fibre enrichment on GIP postprandial secretion [14]. These varying results could once again be attributable to differences between fibre types and doses, food matrices and type of intervention (short vs. long term).

In our study, we observed that intake of a single lower dose of fibre-enriched orange juice did not significantly modify the area under the glucose and insulin curves, but did lead to a significant, yet transient, reduction in serum glucose and insulin levels in healthy individuals when compared with intake of a regular orange juice. These results are in line with other studies, such as that by Huang et al., who observed a reduction solely in the glycaemia response of healthy subjects (5.0 g enzyme-treated orange pomace fibre) [19], and that by Chen et al., who reported decreased glycaemic and insulinemic responses in overweight men, but only when the fibre content of orange pomace (as part of a co-consumed breakfast) reached a certain level (5.48 vs. 2.55 g) [21]. The mechanisms of action of this response and its health implications warrant further research, though the authors hypothesised that fibre slows gastric emptying, thus prolonging glucose digestion and absorption in the upper GI tract, and also interfering with glucose intestinal absorption. Furthermore, this reduction in glycaemia and insulin response 15 min after intake was unexpectedly accompanied by a significant decrease in GLP1 postprandial levels in the intervention group. This result is similar to that reported by Juvonen et al. [37], who observed that high viscous fibre levels diminished the release of anorexigen hormones like GLP1 in relation to low viscous fibre. Consequently, the high viscosity of our test beverage may have impeded the interaction between glucose and the intestinal mucosa, thus delaying both glucose absorption and GLP1 release with respect to the placebo group. 

In relation to other gastrointestinal hormones such as PYY, ghrelin and GIP, we noted a rising trend in the levels of PYY and GIP after fibre-enriched orange juice intake (and a decreasing one in the case of ghrelin), but we did not observe significant differences between the two groups. In contrast to other studies, our intervention was acute and hormone levels were evaluated without the intake of a later meal. Moreover, the release of GI hormones like PYY and ghrelin is mediated by the amount of energy in a meal [33]; our product contained approximately 200 kcal, which is under that of a typical meal, and thus may have been insufficient to produce a significant release of both peptides. On the other hand, leptin is a hormone that is primarily produced by adipocytes and can reduce appetite sensation in the central nervous system. Studies have analysed how different types of diets can affect leptin levels, mainly due to sugar and fibre content, but the evidence that fibre supplements increase leptin levels is not strong [9]. Our fibre-enriched orange juice did not significantly modify leptin levels, probably due to leptin secretion being affected primarily by longer-term changes in body composition, such as fat mass index and energy homeostasis [30], thus indicating that acute interventions do not affect its levels.

Finally, the high content of flavonoids in orange could also have affected our results. It is known that some fruit flavonoids can modify cardiovascular risk factors and satiety sensation and affect carbohydrate digestion [7,38] and lipid metabolism [39]. Although the content of flavonoids would have been present in both placebo and fibre-enriched beverages, flavonoid content was not measured, and thus we cannot assure that it was not modified by fibre-enrichment. Apart from flavonoids, micronutrient content could also have modified our study parameters. It has been shown that vitamin C content can influence glucose and insulin levels [40]. Although the orange juice matrix we used was the same for test and placebo beverages, the juice with the added fibre underwent agitation and homogenization treatments to disperse the fibre, which would have led to a certain loss in vitamin C content. In light of this, more studies are needed to investigate the effects of flavonoids and micronutrients on our study parameters. 

A strength of this study is that we have employed a randomised, placebo-controlled, cross-over study design. Moreover, we have used a novel by-product of orange juice production that is rich in both soluble and insoluble fibre (not enzyme-hydrolysed), which we used to supplement the same matrix. In addition, we performed regular blood sampling to closely evaluate glycaemic and insulin responses, GI hormone response and satiety feelings over 120 min. On the other hand, we should point out that our study involved a relatively small, though homogenous, sample of healthy subjects with equal sex distribution, and a within-subject crossover study design was applied to increase the power of the study. A limitation of the study is that all the participants were relatively young, so the results of the study cannot necessarily be extrapolated to an older population. Although the mechanism of satiety is complex and there are interacting factors such as gastric emptying time, digestibility, volume, the presence of other nutrients, we minimised these errors by using a base matrix with the same nutritional composition and volume. According to the preliminary test performed by our group, 3.5 g was the maximal amount of fibre that could be incorporated into a serving while maintaining an acceptable texture and taste. With this study, we have proved that large amounts of fibre (> 5 g fibre per serving) are not required to observe a significant effect on satiety and postprandial glycaemic response in healthy subjects but probably are needed to trigger a significant reduction of both glucose and insulin AUC responses. We cannot be sure if the metabolic changes we observed would be maintained over longer periods of time and at different doses, in our subjects, or in other individuals with an impaired glucose metabolism. In addition, the polyphenols present in this by-product of orange juice may have influenced postprandial glycaemic responses. Consequently, further research into the long-term impact of fibre-enriched orange juice on GI hormones consumed as part of a regular meal is warranted.

## 5. Conclusions

In summary, our findings demonstrate the beneficial effect-in the initial phase of absorption-of a single acute dose of fibre-enriched orange juice on glucose and insulin levels in healthy individuals with normal weight compared with a placebo. In addition, juice enriched in fibre has a significant effect on satiety and fullness. This is accompanied by a decrease in the levels of GLP1, a peptide that stimulates the secretion of insulin at the duodenal level. 

The dietary intervention employed in this study would appear to be an effective first-step strategy for improving satiety and early postprandial glycaemic response, although further research is necessary to assess whether chronic doses of fibre are beneficial in subjects with altered glycaemia.

## Figures and Tables

**Table 1:** Nutritional composition of the orange juice administered in this study (expressed in g/100 mL).

	Fibre-Enriched Orange Juice	Placebo
Energy value(Kcal /KJ)	47/197	47/198
Fat (g)	0.07	0.11
Carbohydrates (g)	10.2	10.6
Sugars (g)	9.5	9.7
Glucose (g)	2.4	2.5
Fructose (g)	2.5	2.5
Lactose (g)	< 0.4	< 0.4
Maltose (g)	< 0.4	< 0.4
Sacarose (g)	4.6	4.7
Dietary fibre (g)	1.4	0.17

**Table 2:** Nutritional composition of the citrus fibre extract in the enriched beverage (expressed in g/100 g).

	Orange Fibre (g/100g fibre)
Moisture content	7.3
Non-fibre Carbohydrates (g)	10.6
Sugars (g)	9.3
Total dietary fibre (g)	71.2
Insoluble fibre(g)	41.9
Soluble fibre(g)	29.3

**Table 3:** Anthropometric characteristics of the study participants.

Variables	
N (male/female)	10 (5/5)
Age (years)	29.6 ± 1.8
BMI (Kg/m^2^)	22.7 ± 0.5
Weight (Kg)	67.4 ± 3.0
Height (cm)	172.0 ± 3.4
Waist (cm)	74.3 ± 1.9
Hip (cm)	95.7 ± 1.5
Heart Rate (beats/min)	64.1 ± 4.2
Systolic BP (mmHg)	117.5 ± 3.0
Diastolic BP (mmHg)	71.9 ± 2.2

The data are expressed as mean ± standard error.

## Abbreviations

BMI	Body mass index
GI	Gastrointestinal
GIP	Gastric inhibitory polypeptide
GLP1	Glucagon-Like Peptide 1
HOMA-IR	Homeostatic model assessment of insulin resistance
PYY	Peptide YY
SEM	Standard error of the mean
UHPH	Ultra High Pressure Homogenization
VAS	Visual analogue scale

## References

[1] Dhingra D., Michael M., Rajput H., Patil R.T. (2012). Dietary fibre in foods: A review. J. Food Sci. Technol..

[2] Wei B., Liu Y., Fang Y., Cui J., Wan J. (2018). Dietary fiber intake and risk of metabolic syndrome: A meta-analysis of observational studies. Clin. Nutr..

[3] Stephen A.M., Champ M.M., Cloran S.J., Fleith M., van Lieshout L., Mejborn H., Burley V.J. (2017). Dietary fibre in Europe: Current state of knowledge on definitions, sources, recommendations, intakes and relationships to health. Nutr. Res. Rev..

[4] Hervik A.K., Svihus B. (2019). The Role of Fiber in Energy Balance. J. Nutr. Metab..

[5] Tremblay A., Pérusse L. (2015). Nutrients, satiety and control of energy intake. Appl. Physiol. Nutr. Metab..

[6] Ho I.H., Matia-Merino L., Huffman L.M. (2015). Use of viscous fibres in beverages for appetite control: A review of studies. Int. J. Food Sci. Nutr..

[7] Dong H., Sargent L.J., Chatzidiakou Y., Saunders C., Harkness L., Bordenave N., Rowland I., Spencer J.P., Lovegrove J.A. (2016). Orange pomace fibre increases a composite scoring of subjective ratings of hunger and fullness in healthy adults. Appetite.

[8] Guérin-Deremaux L., Pochat M., Reifer C., Wils D., Cho S., Miller L.E. (2011). The soluble fiber NUTRIOSE induces a dose-dependent beneficial impact on satiety over time in humans. Nutr. Res..

[9] Sánchez D., Miguel M., Aleixandre A. (2012). Dietary fiber, gut peptides, and adipocytokines. J. Med. Food.

[10] Ye Z., Arumugam V., Haugabrooks E., Williamson P., Hendrich S. (2015). Soluble dietary fiber (Fibersol-2) decreased hunger and increased satiety hormones in humans when ingested with a meal. Nutr. Res..

[11] Rahman S., Zhao A., Xiao D., Park E., Edirisinghe I., Burton-Freeman B.M. (2017). A Randomized, Controlled Trial Evaluating Polydextrose as a Fiber in a Wet and Dry Matrix on Glycemic Control. J. Food Sci..

[12] Tarini J., Wolever T.M. (2010). The fermentable fibre inulin increases postprandrial serum short-chain fatty acids and reduces free-fatty acids and ghrelin in healthy subjects. Appl. Physiol. Nutr. Metab..

[13] Verhoef S.P., Meyer D., Westerterp K.R. (2011). Effects of oligofructose on appetite profile, glucagon-like peptide 1 and peptide YY3-36 concentrations and energy intake. Br. J. Nutr..

[14] Barone-Lumaga R., Azzali D., Fogliano V., Scalfi L., Vitaglione P. (2012). Sugar and dietary fibre composition influence, by different hormonal response, the satiating capacity of a fruit-based and a β-glucan-enriched beverage. Food Funct..

[15] Rahat-Rozenbloom S., Fernandes J., Cheng J., Wolever T.M.S. (2017). Acute increases in serum colonic short-chain fatty acids elicited by inulin do not increase GLP-1 or PYY responses but may reduce ghrelin in lean and overweight humans. Eur. J. Clin. Nutr..

[16] Jenkins D.J., Wolever T.M., Leeds A.R., Gassull M.A., Haisman P., Dilawari J., Goff D.V., Metz G.L., Alberti K.G. (1978). Dietary fibres, fibre analogues, and glucose-tolerance: Importance of viscosity. Br. Med. J..

[17] Maki K.C., Carson M.L., Miller M.P., Turowski M., Bell M., Wilder D.M., Rains T.M., Reeves M.S. (2008). Hydroxypropylmethylcellulose and methylcellulose consumption reduce postprandrial insulinemia in overweight and obese men and women. J. Nutr..

[18] Thompson S.V., Hannon A., An R., Holscher H.D. (2017). Effects of isolated soluble fiber supplementation on body weight, glycemia, and insulinemia in adults with overweight and obesity: A systematic review and meta-analysis of randomized controlled trials. Am. J. Clin. Nutr..

[19] Huang Y., Park E., Replogle R., Boileau T., Shin J.E., Burton-Freeman B.M., Edirisinghe I. (2019). Enzyme-treated orange pomace alters acute glycemic response to orange juice. Nutr. Diabetes.

[20] Dong H., Rendeiro C., Kristek A., Sargent L.J., Saunders C., Harkness L., Rowland I., Jackson K.G., Spencer J.P., Lovegrove J.A. (2016). Addition of Orange Pomace to Orange Juice Attenuates the Increases in Peak Glucose and Insulin Concentrations after Sequential Meal Ingestion in Men with Elevated Cardiometabolic Risk. J. Nutr..

[21] Chen C.O., Rasmussen H., Kamil A., Du P., Blumberg J.B. (2017). Orange Pomace Improves Postprandrial Glycemic Responses: An Acute, Randomized, Placebo-Controlled, Double-Bind, Crossover Trial in Overweight Men. Nutrients.

[22] Li S., Guérin-Deremaux L., Pochat M., Wils D., Reifer C., Miller L.E. (2010). NUTRIOSE dietary fiber supplementation improves insulin resistance and determinants of metabolic syndrome in overweight men: A double-bind, randomized, placebo-controlled study. Appl. Physiol. Nutr. Metab..

[23] Capuano E. (2017). The behaviour of dietary fibre in the gastrointestinal tract determines its physiological effect. Crit. Rev. Food Sci. Nutr..

[24] Vuksan V., Panahi S., Lyon M., Rogovik A.L., Jenkins A.L., Leiter L.A. (2009). Viscosity of fiber preloads affects food intake in adolescents. Nutr. Metab. Cardiovasc. Dis..

[25] Zijlstra N., Mars M., de Wijk R.A., Westerterp-Plantenga M.S., de Graaf C. (2008). The effect of viscosity on ad libitum food intake. Int. J. Obes..

[26] Lyly M., Ohls N., Lähteenmäki L., Salmenkallio-Marttila M., Liukkonen K.H., Karhunen L., Poutanen K. (2010). The effect of fibre amount, energy level and viscosity beverages containing oat fibre supplement on perceived satiety. Food Nutr. Res..

[27] Ulmius M., Johansson A., Onning G. (2009). The influence of dietary fibre source and gender on the postprandrial glucose and lipid response in healthy subjects. Eur. J. Nutr..

[28] Reimer R.A., Pelletier X., Carabin I.G., Lyon M., Gahler R., Parnell J.A., Wood S. (2010). Increased plasma PYY levels following supplementation with the functional fiber PolyGlycopleX in healthy adults. Eur. J. Clin. Nutr..

[29] Sandberg J.C., Björck I.M.E., Nilsson A.C. (2017). Effects of whole grain rye, with and without resistant starch type 2 supplementation, on glucose tolerance, gut hormones, inflammation and appetite regulation in an 11–14.5 hour perspective; a randomized controlled study in healthy subjects. Nutr. J..

[30] Geliebter A., Grillot C.L., Aviram-Friedman R., Hag S., Yahav E., Hashim S.A. (2015). Effects of oatmeal and corn flakes cereal breakfasts on satiety, gastric emptying, glucose, and appetite-related hormones. Ann. Nutr. Metab..

[31] Lafond D.W., Greaves K.A., Maki K.C., Leidy H.J., Romsos D.R. (2015). Effects of two dietary fibers as part of ready-to-eat cereal (RTEC) breakfasts on perceived appetite and gut hormones in overweight women. Nutrients.

[32] Karhunen L.J., Juvonen K.R., Flander S.M., Liukkonen K.H., Lähteenmäki L., Siloaho M., Laaksonen D.E., Herzig K.H., Uusitupa M.I., Poutanen K.S. (2010). A psyllium fiber-enriched meal strongly attenuates postprandrial gastrointestinal peptide release in healthy young adults. J. Nutr..

[33] Juvonen K.R., Salmenkallio-Marttila M., Lyly M., Liukkonen K.H., Lähteenmäki L., Laaksonen D.E., Uusitupa M.I., Herzig K.H., Poutanen K.S., Karhunen L.J. (2011). Semisolid meal enriched in oat bran decreases plasma glucose and insulin levels, but does not change gastrointestinal peptide responses or short-term appetite in healthy subjects. Nutr. Metab. Cardiovasc. Dis..

[34] Kristensen M., Jensen M.G. (2011). Dietary fibres in the regulation of appetite and food intake. Importance of viscosity. Appetite.

[35] Bodinham C.L., Al-Mana N.M., Smith L., Robertson M.D. (2013). Endogenous plasma glucagon-like peptide-1 following acute dietary fibre consumption. Br. J. Nutr..

[36] Klosterbuer A.S., Thomas W., Slavin J.L. (2012). Resistant starch and pullulan reduce postprandrial glucose, insulin, and GLP-1, but have no effect on satiety in healthy humans. J. Agric. Food Chem..

[37] Juvonen K.R., Purhonen A.K., Salmenkallio-Marttila M., Lähteenmäki L., Laaksonen D.E., Herzig K.H., Uusitupa M.I., Poutanen K.S., Karhunen L.J. (2009). Viscosity of oat bran-enriched beverages influences gastrointestinal hormonal responses in healthy humans. J. Nutr..

[38] Zheng J., Zhou Y., Li S., Zhang P., Zhou T., Xu D.P., Li H.B. (2017). Effects and Mechanisms of Fruit and Vegetable Juices on Cardiovascular Diseases. Int. J. Mol. Sci..

[39] Rampersaud G.C., Valim M.F. (2017). 100% citrus juice: Nutritional contribution, dietary benefits, and association with anthropometric measures. Crit. Rev. Food Sci. Nutr..

[40] Ashor A.W., Werner A.D., Lara J., Willis N.D., Mathers J.C., Siervo M. (2017). Effects of vitamin C supplementation on glycaemic control: A systematic review and meta-analysis of randomised controlled trials. Eur. J. Clin. Nutr..

