# Erratum: Bosch-Sierra, N., et al. Effect of Fibre-Enriched Orange Juice on Postprandial Glycaemic Response and Satiety in Healthy Individuals: An Acute, Randomised, Placebo-Controlled, Double-Blind, Crossover Study. *Nutrients* 2019, *11*, 3014

**DOI:** 10.3390/nu13020696

**Published:** 2021-02-22

**Authors:** Neus Bosch-Sierra, Roger Marqués-Cardete, Aránzazu Gurrea-Martínez, Carmen Grau-Del Valle, Clara Talens, Saioa Alvarez-Sabatel, Carlos Bald, Carlos Morillas, Antonio Hernández-Mijares, Celia Bañuls

**Affiliations:** 1 Service of Endocrinology, University Hospital Doctor Peset, Foundation for the Promotion of Health and Biomedical Research in the Valencian Region (FISABIO), Avda Gaspar Aguilar 90, 46017 València, Valencia, Spain; neusboschsi@gmail.com (N.B.-S.); carmendosaguas@hotmail.com (C.G.-D.V.); carlos.morillas@uv.es (C.M.); antonio.hernandez@uv.es (A.H.-M.); 2 Zumos Valencianos del Mediterraneo S.A., Calle del Pollancar (Pol Industrial Parc Sagunt I) S/N, 46520 Puerto de Sagunto, Valencia, Spain; rmarques@zuvamesa.com (R.M.-C.); agurrea@zuvamesa.com (A.G.-M.); 3 AZTI, Food Research, Basque Research and Technology Alliance (BRTA), Parque Tecnológico de Biz-kaia, Astondo Bidea, Edificio 609, 48160 Derio, Bizkaia, Spain; ctalens@azti.es (C.T.); salvarez@azti.es (S.A.-S.); cbald@azti.es (C.B.); 4 Department of Medicine, University of Valencia, Avda de Blasco Ibañez 15, 46010 València, Valencia, Spain

The authors have requested that the following changes be made to their paper [1].

The production process carried out in the pilot plant to obtain the citrus fibre ingredient used in this work had associated intellectual rights that are the property of AZTI. The original authors therefore wish to add the following three authors from AZTI (Clara Talens, Saioa Alvarez-Sabatel, Carlos Bald) to the paper.

The author’s contribution was: “C.T., S.A.-S. and C.B. (Carlos Bald) produced the fibre and physicochemically characterised the citrus fibre ingredient.”

We would like to apologize for any inconvenience caused to the authors and readers by this mistake. The published version will be updated on the article webpage, with a reference to this notice.
